# Working Together to Build a Better Future for Children With Cancer in Africa

**DOI:** 10.1200/GO.20.00170

**Published:** 2020-07-16

**Authors:** Inam Chitsike, Vivian Paintsil, Lillian Sung, Festus Njuguna, Annelies Mavinkurve-Groothuis, Francine Kouya, Peter Hesseling, Gertjan Kaspers, Glenn M. Afungchwi, Andre Ilbawi, Lorna Renner, Kathy Pritchard-Jones, Laila Hessissen, Elizabeth Molyneux, George Chagaluka, Trijn Israels

**Affiliations:** ^1^ Department of Pediatrics, College of Health Sciences, Harare, Zimbabwe; ^2^ Directorate of Child Health, Komfo Anokye Teaching Hospital, Kumasi, Ghana; ^3^ The Hospital for Sick Children, Toronto, Ontario, Canada; ^4^ Moi University/Moi Teaching and Referral Hospital, Eldoret, Kenya; ^5^ Princess Máxima Center for Pediatric Oncology, Utrecht, the Netherlands; ^6^ Department of Pediatric Oncology, Mbingo Baptist Convention Hospital, Mbingo, Cameroon; ^7^ Department of Pediatrics and Child Health, Stellenbosch University, South Africa; ^8^ Emma Children’s Hospital, Amsterdam UMC, Vrije Universiteit Amsterdam, Pediatric Oncology, Amsterdam, the Netherlands; ^9^ Global Initiative for Childhood Cancer, WHO, Geneva, Switzerland; ^10^ Department of Child Health, University of Ghana Medical School, Accra, Ghana; ^11^ University College London, London, United Kingdom; ^12^ Pediatric Hematology and Oncology Center, Mohamed V University, Rabat, Morocco; ^13^ Department of Pediatrics, College of Medicine, Blantyre, Malawi

There has been substantial improvement in survival of children with cancer in high-income countries. However, great challenges remain in low- and middle-income countries, where > 80% of children with cancer live.^1^ Survival in many countries in Africa, for example, is estimated to be < 20%.^2^ The WHO recently launched the Global Initiative for Childhood Cancer (GICC), which aims to increase survival of children with cancers worldwide to > 60% by 2030 by promoting access to high-quality cancer care for all children and with an initial focus on common and curable cancer types.^3^

As a group of pediatricians caring for children with cancer in Africa, we recognized the need to focus on 3 activities—quality service provision, local data, and locally relevant clinical research—to allow us to improve outcomes together. We realized that service provision would not improve if we continued to rely on fragmented protocol development and outcome assessment at separate units. We required local data to develop locally relevant, collaborative studies to find sustainable solutions to local challenges that will result in substantial and sustained long-term gains. On the basis of these 3 pillars of quality service, local data, and locally relevant research, we are committed to coordinate research to establish and promote best practices within our network.

Oncology units in well-resourced settings have benefit-ted greatly from multicenter collaborations in all aspect of cancer care delivery, resulting in improved outcomes for children with cancer. Multicenter collaborations can and should be global in their design and value.^4^ Accurate local data on numbers of patients on treatment, accuracy of diagnosis, causes of treatment failure, and the efficacy of specific interventions are required to in-form the strategies for improved care and outcomes. Our aim is to increase the survival of children with common and curable cancers in Africa to exceed 60%, in line with the WHO GICC.

With the same goal in mind, the Collaborative Wilms Tumor Africa project was formed in 2014 and has been implementing a consensus-adapted treatment guideline in 8 centers in sub-Saharan Africa as a multicenter clinical trial.^5^ This guideline was developed by the Committee for Pediatric Oncology in Developing Countries (PODC) of the International Society of Paediatric Oncology (SIOP).^6,7^ Currently participating centers are in Blantyre (Malawi); Eldoret (Kenya); Accra and Kumasi (Ghana); Mbingo, Banso, and Mutengene (Cameroon); and Harare (Zimbabwe). Funding was received from SIOP and World Child Cancer and distributed to all participating centers to cover treatment, travel, and other associated costs for patients.

In the first 4 years of the trial (2014-2018), 201 patients were included. After implementation, compared with the baseline evaluation, survival without evidence of disease at the end of treatment increased (69% *v* 52%, respectively; *P* = .002), abandonment of treatment declined (12% *v* 23%, respectively; *P* = .009), and fewer patients died during treatment (13% *v* 21%, respectively; *P* = .06).^8^ Two-year event-free survival was 49.9% ± 3.8% in this patient cohort when abandonment of treatment was considered an event.^9^ The Collaborative Wilms Tumor Africa Project Phase II is planned to start in the second half of 2020 and aims to improve survival further. There are minor revisions to the comprehensive adapted treatment guide-line based on lessons learned in Phase I.^10^

After establishing, implementing, and evaluating initial treatment guidelines, we analyzed clinical data and recognized that the major barrier to using more intensive treatment regimens was the lack of optimal supportive care.^9^ In 2019, Supportive Care for Children With Cancer in Africa (SUCCOUR) was initiated, with a goal to improve supportive care and reduce treatment-related mortality further. Building on the regional network of the Collaborative Wilms Tumor Africa Project and the lessons learned, SUCCOUR aims to promote improvements in supportive care for every child in Africa to be able to be cured from cancer.

We are currently conducting a baseline evaluation of current practices and outcomes in the following important areas of supportive care: malnutrition, febrile neutropenia, abandonment, and treatment-related mortality. This baseline evaluation will be fundamental to understanding the current situation and will facilitate the development and prioritization of supportive care interventions. It will provide a benchmark for future evaluation of the impact of implemented interventions.

Abandonment, or incomplete treatment, is known to be an important problem in our centers, and our goal will be to reduce it to zero. Hence, the third project, Toward Zero Percent Abandonment, was started in Blantyre, Malawi, in 2019. It aims to eliminate incomplete treatment. Abandonment of treatment is a known common and preventable cause of treatment failure in low-income countries.^11^ The baseline evaluation of this project in Malawi documented that 49 (19%) of 264 patients diagnosed in 2018 and 2019 with common and curable cancers had abandoned treatment.^12^ Interventions in Malawi to prevent abandonment include full coverage of treatment, accommodation and transport costs, a tracking system to remind patients of appointments, and more systematic and improved counseling of parents of the need to complete treatment. We intend to introduce this project in all participating centers over the next 1-3 years.

The Collaborative Wilms Tumor Africa Project, SUCCOUR, and Toward Zero Percent Abandonment form the current core of the Collaborative African Network of Clinical Care and Research for Childhood Cancer (CANCaRe Africa). We see the network as a platform on which to build these collaborative studies with the intention that other studies can and will be added to create change. The vision of CANCaRe Africa is that children with common and curable cancers in sub-Saharan Africa will achieve survival rates > 60%-70%, in line with the GICC. The mission is to achieve this by reducing treatment-related deaths to < 10%; reducing abandonment of treatment to < 10%; and developing, implementing, and evaluating locally appropriate treatment guidelines.

CANCaRe Africa has collaborated and aligned with other national and international initiatives. We are sharing our best practices with the WHO GICC, allowing our platform, experience, and knowledge to serve the broader community. Collaborative research and innovation are essential to achieving the targets established by the WHO initiative, and we are actively supporting these WHO workstreams. Collaborative research and innovation are essential to achieving the targets established by the WHO GICC; we are actively supporting WHO workstreams related to establishing treatment standards, to incorporating supportive care programs as part of universal health coverage, and to defining core indicators used in monitoring programs and research. SIOP, including SIOP Africa and SIOP PODC, is the global scientific pediatric oncology umbrella organization.^13^ We collaborate within the SIOP community to learn from other groups, develop our guidelines, and implement and evaluate them according to robust scientific standards. We also work closely with our national governments, allowing for sustainable uptake of our programs in the public sector. Current funding for the activities of this regional network comes from SIOP, World Child Cancer, and the Sanofi Espoir Foundation, who share our vision. We hope to expand these partnerships and include others to have sustainable and hopefully increased funding to deliver on our aims and objectives.

Over the past few years, we have learned many lessons.^14^ We do work according to a shared vision, mission, and principles by designing feasible interventions, achieving incremental steps, and ensuring long-term sustained impact. We give priority to interventions with the highest expected impact on child survival, cognizant of the current profound inequalities. Our philosophy is that local leaders are in the best position to assess feasibility of interventions and set priorities. Transparency, trust, and a shared purpose are essential. We work through national institutional review boards, ensure the validity of our results, and routinely report successes and challenges internally and externally. Friendship, good communication, and comradery are facilitators in achieving our vision and at the core of our success.

We have established an active regional network for childhood cancer that can be a platform for further initiatives to improve care and survival, including but not limited to the WHO GICC. This is only the beginning of our work. Together, we are finding sustainable solutions to shared local challenges for children with cancer in our community and around the world.
